# 858. Diagnostic Accuracy of Monocyte Distribution Width as a Sepsis Biomarker in a Large, Urban Emergency Department

**DOI:** 10.1093/ofid/ofad500.903

**Published:** 2023-11-27

**Authors:** Jordan Crew, Ben Hunter, Armisha Desai

**Affiliations:** Indiana University Health Methodist Hospital, NOBLESVILLE, Indiana; Indiana University Health Methodist Hospital, NOBLESVILLE, Indiana; Indiana University Health Adult Academic Health Center, Indianapolis, IN

## Abstract

**Background:**

Sepsis is a leading cause of hospital and emergency department (ED) mortality, and early identification is crucial. Procalcitonin (PCT), C-reactive protein (CRP), and erythrocyte sedimentation rate (ESR) are increasingly being utilized to aid in the early identification of septic patients. Recently, monocyte distribution width (MDW), a measure of the range in size of monocytes in the blood, has shown promise as an additional sepsis biomarker; levels > 20 have been correlated with an increased risk of sepsis. This investigation sought to determine the diagnostic accuracy of MDW as a marker of sepsis, and compare the accuracy to that of PCT, CRP, and ESR.

**Methods:**

This is a retrospective chart review of adult ED patients from 4/1/2022 to 7/31/2022 in whom an MDW was resulted. During this time, MDW was included as part of all CBC reports. We collected PCT, CRP, and ESR results if obtained within 48 hours of the MDW. Sepsis diagnoses were based on a primary ICD-10 diagnosis of sepsis (A40-A41) at ED or hospital discharge. Sensitivity, specificity, positive predictive value (PPV), and negative predictive value (NPV) were calculated for MDW in all patients, MDW only in patients with PCT also ordered (regardless of PCT result), PCT, CRP+ESR, and MDW+PCT for the sake of comparison. MDW was considered “positive” for sepsis if > 20.

**Results:**

There were 10,654 patient encounters with MDW reported. The mean age was 51 years old, and 55% of patients were female. Of the 10,654 encounters, 269 (2.5%) had a sepsis diagnosis on discharge. Diagnostic test results for MDW, PCT, and ESR and CRP are displayed in Table 1. Among all ED patients with a CBC, MDW had a sensitivity, specificity, PPV, and NPV of 82%, 69%, 6.7% and 99%, respectively. In 847 patients where infection was being considered (based on the independent ordering of PCT), MDW sensitivity, specificity, PPV, and NPV were 85%, 53%, 29% and 94%, respectively. MDW was more sensitive but less specific than PCT. In cases of both positive MDW and PCT, the PPV for sepsis was 43%.

**Figure:**
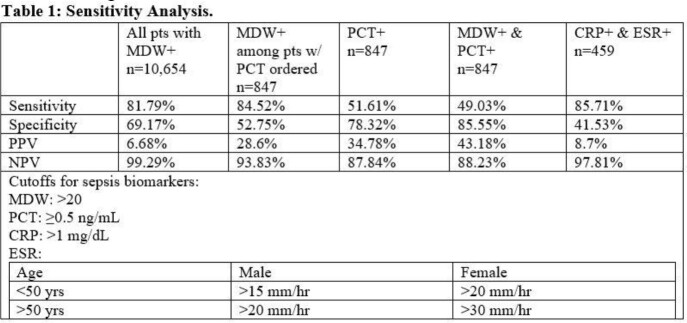

**Conclusion:**

These findings suggest that, as a marker for sepsis, MDW has comparable test characteristics to ESR and CRP, with higher sensitivity but lower specificity than PCT. The combination of a positive MDW and PCT was most predictive of sepsis.

**Disclosures:**

**Ben Hunter, MD**, Beckman Coulter: Honoraria

